# Impact of Deficiency of Intrinsic Coagulation Factors XI and XII on Ex Vivo Thrombus Formation and Clot Lysis

**DOI:** 10.1055/s-0039-1693485

**Published:** 2019-09-10

**Authors:** José W. P. Govers-Riemslag, Joke Konings, Judith M. E. M. Cosemans, Johanna P. van Geffen, Bas de Laat, Johan W. M. Heemskerk, Yesim Dargaud, Hugo ten Cate

**Affiliations:** 1 Department of Biochemistry, Cardiovascular Research Institute Maastricht (CARIM), Maastricht University Medical Center, Maastricht, The Netherlands; 2 Department of Internal Medicine, Cardiovascular Research Institute Maastricht (CARIM), Maastricht University Medical Center, Maastricht, The Netherlands; 3 Synapse Research Institute, Cardiovascular Research Institute Maastricht (CARIM), Maastricht University, Maastricht, The Netherlands; 4 Unité d ‘Hémostase Clinique, Hôpital Cardiologique Louis Pradel, Hospices Civils de Lyon, Bron, France

**Keywords:** clot lysis, factor XII, factor XI, deficiency, thrombus formation

## Abstract

The contributions of coagulation factor XI (FXI) and FXII to human clot formation is not fully known. Patients with deficiency in FXI have a variable mild bleeding risk, whereas FXII deficiency is not associated with bleeding. These phenotypes make FXII and FXI attractive target proteins in anticoagulant therapy. Here, we studied the mechanisms of fibrin clot formation, stability, and fibrinolytic degradation in patients with severe FXI or FXII deficiency. Thrombin generation was triggered in platelet-poor (PPP) and platelet-rich plasma (PRP) with the biological FXII trigger sulfatides. Intrinsic and extrinsic thrombus formation and degradation in whole blood were determined with rotational thromboelastometry (ROTEM). Clot formation under flow was assessed by perfusion of whole blood over collagen microspots with(out) tissue factor (TF). Thrombin generation and clot formation were delayed in FXII- and FXI-deficient patients triggered with sulfatides. In FXI-deficient plasma, this delay was more pronounced in PRP compared to PPP. In whole blood of FXII-deficient patients, clots were smaller but resistance to fibrinolysis was normal. In whole blood of FXI-deficient patients, clot formation was normal but the time to complete fibrinolysis was prolonged. In flow chamber experiments triggered with collagen/TF, platelet coverage was reduced in severe compared with moderate FXI deficiency, and fibrin formation was impaired. We conclude that quantitative defects in FXII and FXI have a substantial impact on contact activation-triggered coagulation. Furthermore, FXI deficiency has a dose-dependent suppressing effect on flow-mediated and platelet/TF-dependent clot formation. These last data highlight the contribution of particularly FXI to hemostasis.

## Introduction

Thrombosis is a critical element in the pathogenesis of ischemic cardiovascular disease as well as in venous thromboembolism. Both epidemiological and animal studies indicate that the intrinsic coagulation factors, factor XI (FXI) and factor XII (FXII), contribute to the development of pathological thrombus formation. 1 2 3 4 In mice, congenital deficiency of either *F11* or *F12* protected against experimentally induced arterial thrombosis. 5 6 7 Since these deficiencies are not associated with bleeding (FXII) or with a mild bleeding phenotype (FXI), respectively, the contribution of these intrinsic factors in normal hemostasis is limited. Moreover, antibody-, antisense-, or aptamer-based inhibitors of FXI or FXII showed promising, suppressive effects in several in vivo models of arterial thrombosis. 8 9 10 11 12 13 Also, in primate thrombosis models, immunological inhibition of FXI or FXII appeared to be thromboprotective. 10 Limited and also conflicting information is available on how reduced levels of FXI and FXII can protect against thrombosis in man, since subjects with a congenital deficiency in either factor still experience cardiovascular events. 14 A recent trial indicated that antisense FXI treatment reduced postoperative venous thromboembolism without increasing bleeding in patients undergoing knee replacement surgery. 15 Although the collective data point to a more prominent role of human FXI and FXII in thrombosis than in hemostasis, it is important to better understand the mechanisms and processes that are regulated by these contact activation factors.

The thrombus formation process involves different pathways that interact with FXI and FXII. Next to a relatively slow triggering via the intrinsic FXII pathway, thrombin generation is potently triggered by the fast, extrinsic tissue factor (TF)/FVIIa pathway. First traces of formed thrombin then feedback to activate platelets and proteolyse coagulation (co)factors, including FXI. The latter process leads to a gradual build-up of sufficiently high thrombin levels to support formation of a fibrin clot. 16 Activated, phosphatidylserine-exposing platelets enhance these reactions to promote both thrombin generation and fibrin formation. Furthermore, there are indications for cross-talk between the intrinsic and extrinsic coagulation pathways, with FXI as an essential element, but under which conditions these become prominent is unclear. Recent evidence also suggests that FXI can be activated on activated platelets, independently of FXII. 17 18 Moreover, FXIIa can interact with fibrin(ogen) resulting in a tighter clot structure and altered fibrinolysis. 19 20 21

The aim of this study, in patients with a congenital deficiency in one of the contact system proteins, is to assess the contribution of FXI and FXII in (patho)physiologically relevant integrative measurements of coagulation activity, i.e., thrombin and fibrin clot generation in plasma and blood under stasis, formation of a fibrin-thrombus under flow conditions, and the degradation of formed clots.

## Material and Methods

### Patients and Healthy Subjects

Included in the study were well-characterized patients with established deficiencies in FXI or FXII and healthy control donors, who were examined in parallel. The study population concerned six patients with FXI deficiency and four patients with FXII deficiency, along with 10 controls subjects of comparable age. The study was designed and performed according to the Helsinki declaration and each subject provided written informed consent. The study was approved by the Lyon University Hospital's medical ethics committee under number ID-RCB 2013-A01274-41.

Overall characteristics of patients and control subjects are indicated in Table 1 . Of the FXI-deficient patients, four had a severe (FXI ≤ 5 IU/dL), one a moderate (FXI 6–15 IU/dL), and one a mild (FXI > 15 and < 60 IU/dL) deficiency. All FXII-deficient patients had a severe deficiency (FXII ≤ 5 IU/dL). All patients had initially been identified based on a prolonged activated partial thromboplastin time (aPTT). None of the FXII-deficient patients had experienced any bleeding or thrombosis problems. Two of the six FXI-deficient patients (1 and 4 IU/dL) had experienced major bleeding, three of the others had a mild bleeding tendency, and one FXI-deficient patient bruises easily. The 10 healthy controls had no personal history of bleeding or thrombosis. Subjects had not received medication affecting platelets or coagulation, including oral contraceptives or anti-inflammatory drugs (NSAIDs), at least during 14 days before blood collection.

**Table 1:** General characteristics of the study population

	Healthy individuals	FXI DEF patients	FXII DEF patients
	( *n* = 10)	( *n* = 6)	( *n* = 4)
Gender (female, %)	80%	50%	25%
Age (years, mean ± SD)	48 ± 10	47 ± 17	34 ± 16
PT (s) [12–14] a	–	13.0 [13.0–15.6]	13.0 [13.0–13.3]
aPTT (s) [28–35] a	–	66 [46–282] b	190 [72–251] b
FXI (IU/dL)	96 [86–124]	3 [<1 to 22] b c	78 [73–125]
FXII (IU/dL)	101 [60–141]	100 [93–108]	2 [<1 to 2] b c
Prothrombin (IU/dL)	95 [85–110]	102 [89–124]	98 [85–99]
Fibrinogen (g/L)	3.2 [2.2–4.1]	3.6 [2.3–4.1]	3.1 [2.4–4.0]
AT (IU/dL)	113 [102–134]	110 [96–115]	101 [96–112]
FXIII activity (IU/dL)	68 [58–147]	79 [56–85]	87 [83–147]
Plasminogen activity (IU/dL)	101 [66–127]	112 [100–135]	97 [86–131]
tPA activity (IU/mL)	0.5 [0.5–0.7]	0.5 [0.4–0.5]	0.6 [0.5–0.7]
PAI-1 activity (ng/mL)	1.5 [1.3–2.0]	1.5 [1.4–2.4]	1.4 [1.3–4.9]
Alpha-2 antiplasmin (IU/dL)	99 [45–136]	85 [65–130]	102 [85–131]
Platelets in PRP (10 ^9^ /L)	309 [191–509]	303 [231–410]	317 [197–429]

Abbreviations: aPTT, activated partial thromboplastin time; AT, antithrombin; DEF, deficient; FXI, factor XI; FXII, factor XII; PAI-1, plasminogen activator inhibitor-1; PRP, platelet-rich plasma; PT, prothrombin time; SD, standard deviation; tPA, tissue plasminogen activator.

Note: Data are presented as median and range [min–max] except for gender and age. *p* -Values were calculated for factor deficiency vs. control (Kruskall–Wallis ANOVA).

a Reference values.

b Indicates that there is a significant difference between deficient patients and controls for the particular parameter ( *p* < 0.05).

c Individual FXI levels in FXI-deficient patients: <1, <1, 2, 4, 6, 22 IU/dL and individual FXII levels in FXII-deficient patients: <1, <1, 2, 2 IU/dL.

### Blood Collection and Processing

Blood was collected into S-Monovette tubes (Sarstedt, Nümbrecht, Germany) containing 0.106 mol/L trisodium citrate in the absence and presence of corn trypsin inhibitor (CTI, Haematologic Technologies, Essex Junction, Vermont, United States; 1.45 μmol/L, final concentration). The first collection tube was discarded. Whole blood rotational thromboelastometry (ROTEM) was performed directly after blood drawing. Platelet-rich plasma (PRP) was prepared by centrifugation at 150 g for 10 minutes at room temperature. Platelet count was adjusted to 150 × 10 ^9^ /L by dilution with autologous platelet-poor plasma (PPP). The latter was obtained by a second centrifugation step at 2,500 g for 15 minutes at room temperature. Storage of PPP in aliquots was at −80 °C until analysis. For assays performed with low TF levels, the CTI-containing blood was used, in order to exclude contribution of the contact activation pathway. 22 The CTI concentration of 18.5 µg/mL (1.45 µmol/L) was enough to inhibit FXIIa but avoid inhibition of FXIa. 23

### Materials

CTI was from Haematologic Technologies (Essex Junction, Vermont, United States); recombinant TF Innovin was from Dade Behring (Marburg, Germany); sulfatides and bovine serum albumin were from Sigma (St. Louis, Missouri, United States). Purified human FXII and FXI were obtained from Enzyme Research Laboratories (Stago, Leiden, the Netherlands). Recombinant tissue-type plasminogen activator (tPA) was from Boehringer Ingelheim (Alkmaar, the Netherlands). Synthetic phospholipids DOPS, DOPC, and DOPE (1,2-dioleoyl- *sn* -glycero-3-phosphoserine, 1,2-dioleoyl- *sn* -glycero-3-choline, and 1,2-dioleoyl- *sn* -glycero-3-ethanolamine) were from Avanti Polar Lipids (Alabaster, Alabama, United States). Phospholipid vesicles (DOPS/DOPC/DOPE, 20/60/20 mol/mol/mol) were prepared by sonication, as described earlier. 24 Fluorogenic thrombin substrate, Z-Gly-Gly-Arg-aminomethyl coumarin (Z-GGR-AMC), was from Bachem (Bubendorf, Switzerland).

### Coagulation Assays

Prothrombin time, aPTT, and levels of fibrinogen, antithrombin, and coagulation factors were measured on an ACL-TOP 750 system (Werfen, Le Pré-Saint-Gervais, France). The standards, liquid antithrombin (HemosIL), QFA fibrinogen (HemosIL), recombiplastin prothrombin (HemosIL), and von Willebrand factor (VWF; AcuStar) were from Werfen. ELISA kits purchased from Hyphen Biomed (Nodia, Boom, Belgium) were used for activity tests of FXIII and PAI-1 (Zymutest), plasminogen and α2-antiplasmin (Biophen), and tPA (Zymuphen). All activity tests were performed in citrated plasma samples.

### Thrombin Generation Measurements

The calibrated automated thrombogram (CAT) method (Diagnostica Stago, Asnieres sur Seine, France) was used to measure thrombin generation in PRP and PPP using 96-well plates. 25 Briefly, per well 80 µL plasma was added to 20 µL of trigger consisting of sulfatides (1 µmol/L, f.c.) or TF (0.5 pmol/L, f.c.). In the case of PPP, 4 µmol/L (f.c.) phospholipid vesicles were added to the trigger medium. Thrombin generation was started by adding 20 µL of ZGGR-AMC (416 nmol/L) and CaCl _2_ (16.7 mmol/L), and fluorescence accumulation was followed continuously in time. Platelet concentration in PRP was adjusted to 150 × 10 ^9^ /L.

Where described, plasmas were supplemented with purified FXI (6 pmol in 200 µL) or FXII (75 pmol in 200 µL) on top of the residual level of FXI or FXII present in the plasma. To all plasma samples, the same amount of FXI or FXII, respectively, was added leading to an increase of the endogenous concentration with 100 IU/dL.

From the thrombogram curves, the following parameters were obtained: lagtime (min), endogenous thrombin potential (ETP, nmol/L min ^−1^ ), thrombin peak height (nmol/L), time-to-peak (min), and the velocity index (VI, nmol/L min ^−1^ , equaling the thrombin peak height/[time to peak minus lagtime]).

### Whole Blood Thromboelastometry

Thrombus elasticity parameters were determined by ROTEM (TEM International, Munich, Germany) to assess the build-up and lysis of a platelet–fibrin clot in whole blood. Measurements were performed at 37 °C using 300 μL citrate-anticoagulated blood, which was triggered with 4 μmol/L sulfatides (f.c.) and 16 mmol/L CaCl _2_ (intrinsic activation). Alternatively, the extrinsic pathway was triggered with 15 pmol/L TF and 16 mmol/L CaCl _2_ (CTI-containing blood). To measure clot lysis, tPA (125 ng/mL, f.c.) was added to the intrinsic or extrinsic reagent. 26 Final concentrations of sulfatides, TF, and tPA were as optimized from prior dose–response curves. Where indicated, purified FXI or FXII was added to prewarmed blood samples, immediately before testing.

Where indicated, purified FXI (5.3 pmol in 340 µL) or FXII (65 pmol in 340 µL) was added to prewarmed blood samples, immediately before testing. To all blood samples, the same amount of FXI or FXII was added, regardless of the residual level present in whole blood. Assuming a 50% hematocrit, the supplemented amount of FXI or FXII resulted in an increase of the endogenous concentration with 100 IU/dL.

Evaluated parameters derived from thromboelasticity curves were: clotting time (CT), α-angle (rate of clot formation), and maximal clot firmness (MCF). 27 The clot lysis time (CLT 50%) was calculated as the interval from 50% clot formation to 50% clot lysis. Decay curve parameters further provided information on fibrinolysis at a later stage: the difference between lysis-onset time (LOT at 15% reduction of MCF) and lysis time (LT at 90% reduction of MCF); fibrinolysis rate (FR) was defined as 75%/(LT − LOT), which represents the curve decline in % per minute.

### Flow Experiments with Whole Blood

Tendency of thrombus formation was determined by perfusion of recalcified citrated whole blood over a coated glass coverslip using the parallel-plate Maastricht chamber (dimensions flow channel: width 3 mm, depth 50 μm, length 300 mm). 28 Glass coverslips were coated with microspots of collagen type I (applied at 50 µg/mL) with or without 10 pg applied recombinant TF, and used for flow perfusion assays, as described. 29 Blood samples were prelabeled with DiOC _6_ (0.5 μg/mL, f.c., stains mitochondria, vesicle membranes and endoplasmic reticulum of platelets and other cells), the phosphatidylserine probe Alexa Fluor (AF)568-annexin A5 (1:200), and the fibrin precursor (AF)647-fibrinogen (16.5 μg/mL, f.c.). The samples were co-infused with 10 vol% coagulation medium (Hepes buffer pH 7.45 supplemented with 32 mmol/L MgCl _2_ and 63 mmol/L CaCl _2_ ), using two pulse-free pumps. This resulted in physiological (mmol/L) concentrations of free Ca ^2+^ and Mg ^2+^ . Wall-shear rate at the microspots was 1,000 s ^−1^ . Where indicated, purified FXI (5.2 pmol in 1000 µL) was added to blood samples, immediately before testing. To all blood samples, the same amount of FXI was added, regardless of the residual level present in whole blood. Assuming a 50% hematocrit, the supplemented amount of FXI or FXII resulted in an increase of the endogenous concentration with 33 IU/dL.

Brightfield microscopic images were captured at regular time intervals to monitor time-to-first-fibrin formation. After 11 minutes of blood perfusion, two-colored images were recorded using a fast line-scanning confocal Zeiss LSM7 system, equipped with a 63× oil-immersion objective (Carl Zeiss, Oberkochen, Germany). 29 Confocal fluorescence images were analyzed by thresholding and assessment of surface area coverage above threshold, as before. 30 At least five images were taken per microspot/color.

### Statistical Analysis

Statistical analyses were performed with GraphPad Prism for Windows, version 6.0 (GraphPad Software, San Diego, California, United States). Paired data within patients were compared with the paired nonparametric Wilcoxon *t* -test. Parameters of thrombus formation between patients and controls were compared using a Kruskall–Wallis ANOVA with Dunn's post-test. Data are expressed as medians ± interquartile ranges; data from individual patients are shown. *p* -Values < 0.05 were considered to be statistically significant.

## Results

### Characteristics of the FXI- and FXII-Deficient Patients

In Table 1 the general characteristics of the study population are shown. In all 10 adult patients, the PT was normal and the aPTT was prolonged, i.e., outside the normal range. FXI-deficient patients were normal in FXII levels, and vice versa. Plasma levels/activities of prothrombin, fibrinogen, antithrombin, and FXIII were within normal ranges and were comparable for patients and controls; the same applied to fibrinolysis parameters (plasminogen, tPA, PAI-1, and α2-antiplasmin). Since FXI deficiency (but not FXII deficiency) is sometimes associated with low levels of VWF, we determined the Von Willebrand ristocetin cofactor [vWF:RCo] activity in FXI-deficient individuals and healthy controls. The median vWF:RCo activity in FXI-deficient patients was 85 IU/dL (range: min–max, 65–130 IU/dL) and did not differ significantly from healthy controls (89 IU/dL [range: min–max, 49–164 IU/dL]). In addition, VWF activity and platelet counts were normal, thus ruling out quantitative VWF-platelet abnormalities.

### Assessing Impairments in Thrombin Generation Using PRP or PPP

Thrombin-generation profiles were measured in the presence or absence of platelets. To specifically study contact activation we chose sulfatides, a component present in several tissues and compartments of the human body, which stimulates contact activation reactions more rapid than kaolin 31 and generates coagulation activity. TF was used to trigger the extrinsic pathway. The optimal trigger concentrations were predetermined in normal plasmas (data not shown). When triggering plasmas from FXII-deficient patients with sulfatides, all thrombin generation parameters were prolonged (lagtime, time to thrombin peak) or reduced (peak height, ETP, VI), regardless of whether PRP or PPP was used ( Table 2 ). For the plasmas from FXI-deficient patients, only lagtime and time-to-thrombin peak were prolonged and were statistically significant in PRP ( Table 2 ).

**Table 2:** Thrombin generation in platelet-rich plasma and platelet-poor plasma—contact pathway

	Healthy controls	FXI DEF patients	FXII DEF patients a
	( *n* = 10)	( *n* = 6)	( *n* = 4)
**Platelet-rich plasma**			
Lagtime (min)	6.5 [5.6–10.0]	16.8 [9.0–35.7] b	55.1 [17.0–120.0] b
Time to peak (min)	13.5 [9.7–16.3]	26.0 [16.2–60.9] b	77.2 [30.5–120.0] b
Peak height (nM)	126 [94–160]	95 [24–145]	32 [0–65] b
ETP (nM min)	1,502 [1,252–1,612]	1,525 [894–2,069]	280 [0–1340] b
VI (nM/min)	25.5 [8.8–49.8]	9.9 [1.0–26.9] b	1.5 [0–4.9] b
**Platelet-poor plasma**			
Lagtime (min)	6.0 [3.7–9.7]	11.3 [3.7–29.3]	36.2 [12.8–51.3] b
Time to peak (min)	7.8 [5.1–12.2]	15.2 [5.2–37.6]	48.0 [19.4–62.0] b
Peak height (nM)	293 [206–338]	159 [6–398]	8 [6–47] b
ETP (nM min)	1,082 [859–1,236]	1,098 [108–1,531]	170 [138–646] b
VI (nM/min)	160.6 [87.9–252.8]	43.9 [0.8–268.5]	0.7 [0.5–0.7] b

Abbreviations: DEF, deficient; ETP, endogenous thrombin potential; VI, velocity index.

Note: The platelet count was set to 150 × 10 ^9^ /L for platelet-rich plasma and 4 μM phospholipids were added to the initiation mixtures for platelet-poor plasma. Data are presented as median and range [min–max]. *p* -Values were calculated for factor deficiency vs. control (Kruskall–Wallis ANOVA).

a In one FXII-deficient patient no measurable thrombin generation was observed (flat thrombin generation curve) in platelet-rich plasma. Therefore, the lagtime and time to peak were set to the maximum of 120 minutes. The peak height, ETP, and velocity index were set to a minimum of 0.

b Indicates that there is a significant difference between deficient patients and controls for the particular parameter ( *p* < 0.05).

**Fig. 1:**
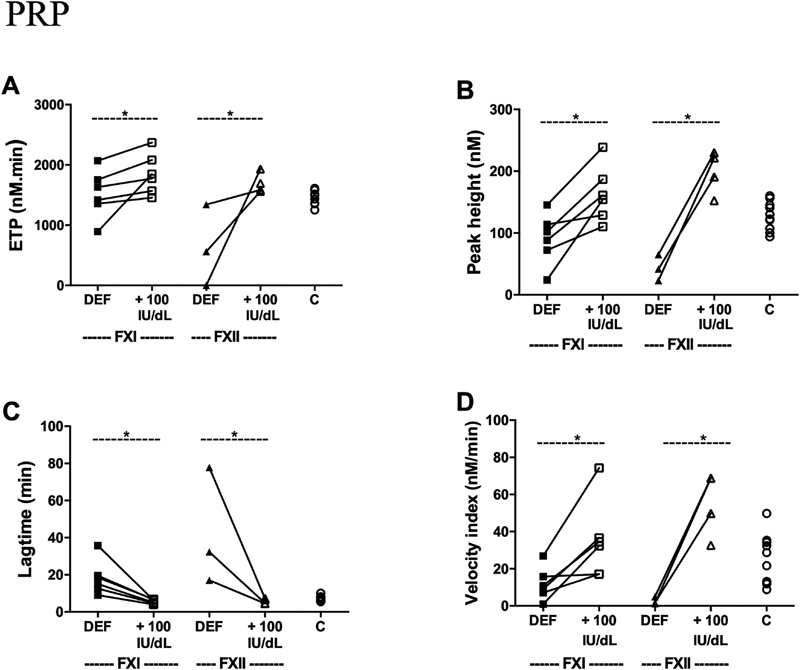
Impaired thrombin generation after triggering the intrinsic pathway in platelet-rich plasma (PRP) from patients improved by factor addition. Thrombin generation in PRP from indicated controls (C) or FXI- or FXII-deficient patients. Triggering was with CaCl _2_ and 4 μmol/L sulfatides. Where indicated, purified FXI (6 pmol in 200 µL) or FXII (75 pmol in 200 µL) was preadded to PRP leading to an increase in the endogenous FXI or FXII concentration of 100 IU/dL. In one FXII-deficient patient, no thrombin generation occurred in PRP without factor XII addition resulting in data *n* = 3 in FXII DEF instead of *n* = 4. The following curve parameters were assessed: ETP (endogenous thrombin potential) ( **A** ), thrombin peak height ( **B** ), lagtime to thrombin generation ( **C** ), and velocity index ( **D** ). * *p* < 0.05 vs. no factor addition (Kruskal–Wallis ANOVA).

In order to check that the changes in the thrombin generation parameters were a consequence of the factor deficiencies, purified FXI or FXII was added to the respective factor-deficient PRP samples, to reach a “normal” level; this process restored thrombin generation parameters upon triggering with sulfatides in PRP from all tested patients ( Fig. 1 ).

### Assessing Impairments in Platelet–Fibrin Elastic Clot Formation

We assessed the formation and lysis of elastic clots consisting of aggregated, contracting platelets, and fibrin using ROTEM. 26 32 Table 3 shows the thromboelastogram parameters upon triggering with sulfatides and TF. Upon triggering with sulfatides, CTs were prolonged (CT, FXI-, and FXII-deficient patients) and curve slopes were reduced (α-angle, FXII-deficient patients) compared to controls. Thromboelastic curve parameters were not affected upon triggering with TF ( Table 3 ). Remarkably, for one of the FXII-deficient patients, thrombolysis parameters after sulfatide stimulation could not be calculated within 2 hours of measurement, because less than 90% of the thrombus was lysed within this time period. We also tested thrombolysis after stimulation of coagulation with TF and for this specific FXII-deficient patient the thrombolysis parameters were detectable but low at the selected concentration of 125 ng/mL tPA. Titration with higher amounts of tPA resulted in a normalized FR in this patient ( Supplementary Fig. S1 ).

**Table 3:** ROTEM parameters—intrinsic and extrinsic pathway

	Sulfatides			TF		
	Healthy controls	FXI DEF patients	FXII DEF patients	Healthy controls	FXI DEF patients	FXII DEF patients
	( *n* = 10)	( *n* = 6)	( *n* = 4)	( *n* = 10)	( *n* = 6)	( *n* = 4)
CT (min)	3.7 [3.2–4.1]	5.1 [3.7–12.7] a	27.9 [17.3–35.9] a	4.8 (2.8–6.7]	3.3 [2.5–4.6]	5.5 [4.8–12.2]
α-angle (deg)	72.9 [64.5–79.0]	67.3 [31.0–75.0]	37.0 [18.0–49.0] a	69.5 [62.0–76.0]	72.5 [63.5–77.5]	68.5 [51.0–71.0]
MCF (mm)	55 [46–62]	49 [32–55]	24 [9–59]	48 [37–60]	52 [41–55]	44 [26–64]
CLT 50% (min)	26.5 [23.1–38.0]	33.3 [25.1–52.1]	24.8 [9.8–51.0]	26.5 [22.6–39.4]	31.0 [26.9–35.5]	25.8 [19.5–80.2]
FR (% decline/min)	5.3 [3.7–7.8]	3.3 [0.9–7.2]	6.2 [4.8–8.0] b	6.0 [2.0–9.2]	3.4 [2.5–6.7]	7.6 [1.4–9.4]

Abbreviations: CT, clotting time; DEF, deficient; MCF, maximum clot firmness; CLT 50%, clot lysis time, interval from 50% clot formation to 50% clot lysis; FR, fibrinolysis rate; TF, tissue factor.

Note: Data are presented as median and range [min–max]. FR was calculated from the ROTEM parameters lysis-onset time (LOT) and lysis time (LT): FR = 75/(LT − LOT) decline in % per minute. *p* -Values: factor deficiency vs. control (Kruskall–Wallis ANOVA).

a Indicates that there is a significant difference between deficient patients and controls for the particular parameter ( *p* < 0.05).

b In one FXII-deficient patient no LT and LOT could be calculated within 120 minutes and therefore no FR could be calculated.

**Fig. 2:**
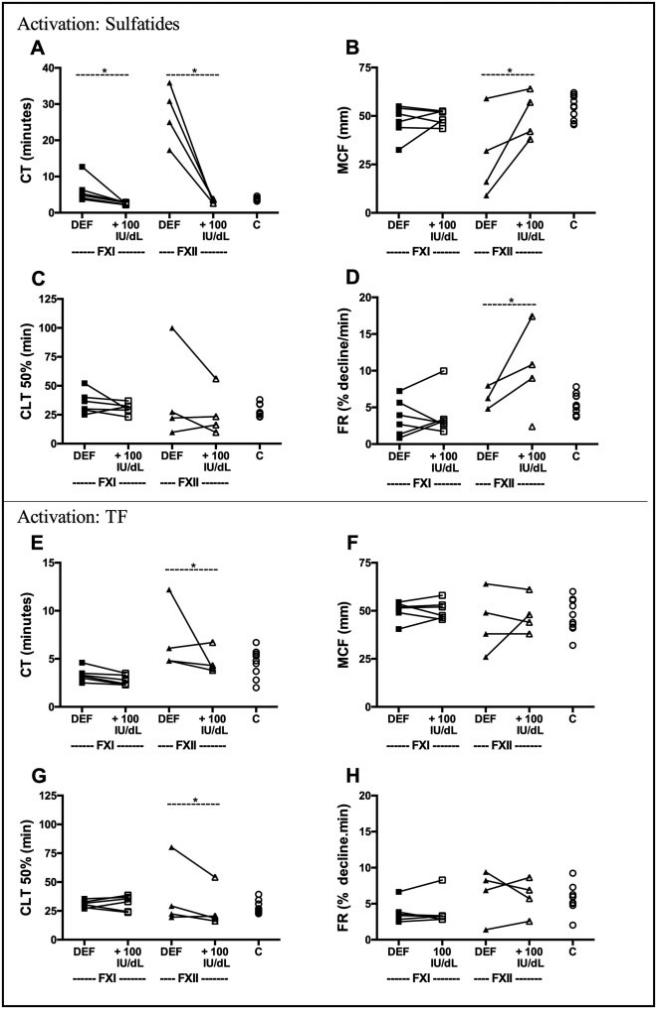
Aberrant elastic clot formation in whole blood from patients normalized by factor addition. ROTEM curves were generated with whole blood from indicated controls (C) or FXI- or FXII-deficient patients. Triggering was with CaCl _2_ and 4 μmol/L sulfatides ( **A–D** ), or CaCl _2_ and 15 pmol/L TF (CTI-treated blood) ( **E–H** ). Where indicated, FXI (5.3 pmol in 340 µL) or FXII (65 pmol in 340 µL) was preadded to the blood leading to an increase in the endogenous FXI or FXII concentration of 100 IU/dL. For one FXII-deficient patient, the fibrinolysis rate after sulfatide stimulation could not be calculated ( **D** ) since lysis velocity was extremely slow and less than 90% of the thrombus was lysed within 2 hours of measurement. The following curve parameters were assessed: CT (clotting time), MCF (maximal clot firmness), CLT (50% clot lysis time), FR (fibrinolysis rate). * *p* < 0.05 vs. no factor addition (Kruskal–Wallis ANOVA). CTI, corn trypsin inhibitor; TF, tissue factor.

To again confirm that any alterations in thromboelastometry curves were linked to the known factor deficiencies, residual patients' blood samples were supplemented with purified FXI or FXII. The added FXI or FXII normalized ROTEM parameters regardless of the trigger ( Fig. 2 ). Of note, in the absence of tPA, no spontaneous clot lysis occurred within 2 hours for all tested blood samples (data not shown).

### Assessing Impairments in Whole Blood Formation of Fibrin–Thrombi under Flow

**Fig. 3:**
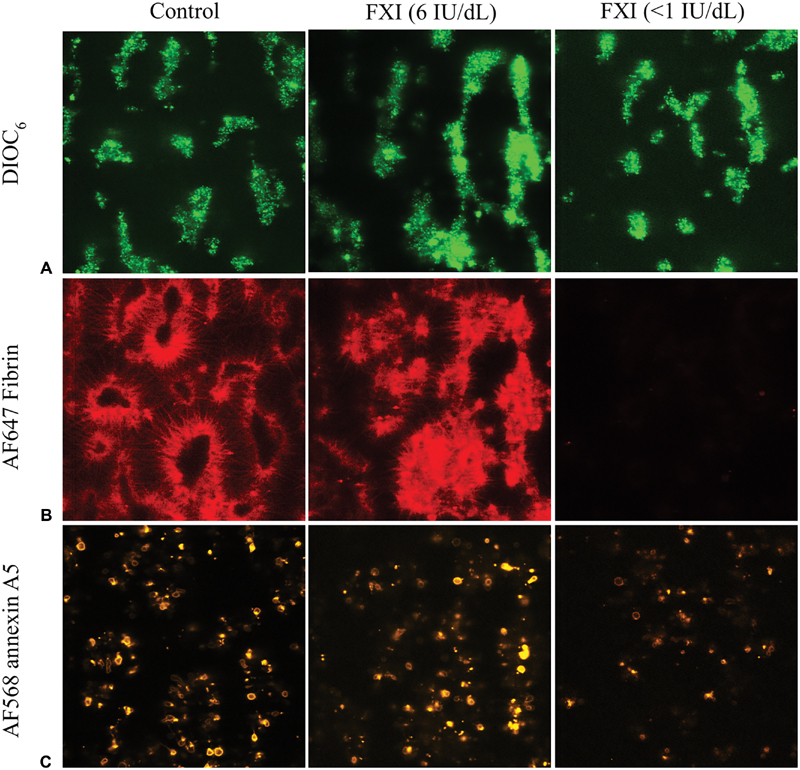
Impaired fibrin-thrombus formation in whole blood from patients with factor XI deficiency. Whole blood samples from representative controls and indicated patients (FXI levels 6 IU/dL and <1 IU/dL, respectively) were perfused under recalcification over microspots of collagen ± TF at a wall-shear rate of 1,000 s ^−1^ for 11 minutes. Real-time recorded brightfield microscopic images were analyzed for fibrin-thrombus formation ( Fig. 4 ). Fluorescence images at endstage were taken for measurement of platelet deposition ( **A** , DiOC _6_ ), AF647-fibrin formation ( **B** ), and phosphatidylserine exposure ( **C** , AF568-annexin A5). Representative images (106 × 106 µm) are shown, captured from microspots of collagen/TF. TF, tissue factor.

**Fig. 4:**
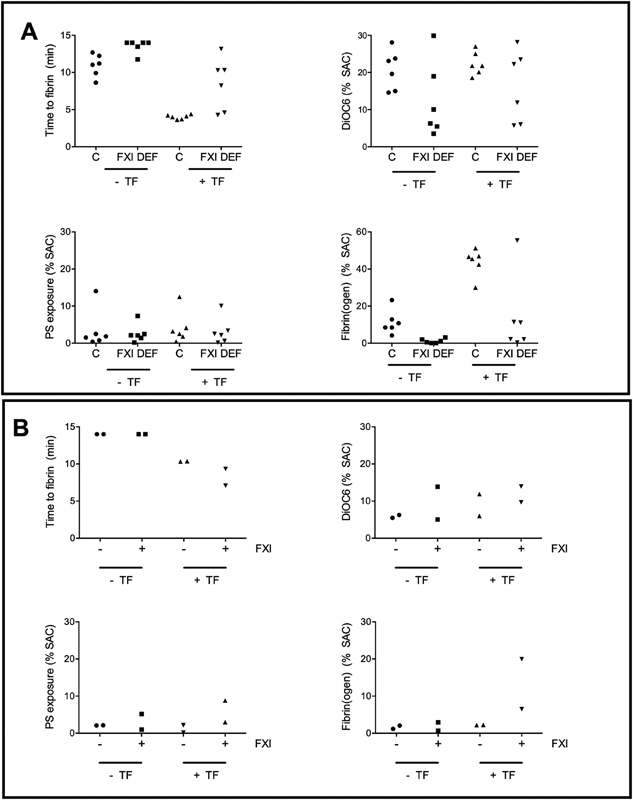
Quantification of fibrin-thrombus formation in whole blood from patients with factor XI deficiency. Whole blood samples from control subjects and indicated FXI-deficient patients were perfused under recalcification over microspots of collagen ± TF for 11 minutes, as described for Fig. 3 . Microscopic images were analyzed for: time-to-first fibrin formation, platelet deposition (DiOC _6_ , %SAC), phosphatidylserine exposure (AF568-annexin A5, %SAC), and AF647-fibrin formation (%SAC) ( **A** ). Furthermore, blood samples from two FXI-deficient patients (4 or <1 IU/dL) were supplemented with purified FXI (5.2 pmol in 1,000 µL) leading to an increase in the endogenous FXI concentration of 33 IU/dL and used for the same perfusion assay. Microscopic images were again analyzed for: time-to-first fibrin formation, platelet deposition (DiOC _6_ ), phosphatidylserine (AF568-annexin A5), and AF647-fibrin formation ( **B** ). TF, tissue factor.

To investigate the process of fibrin–thrombus formation under flow, blood samples from the FXI-deficient patients were preincubated with DiOC _6_ to label platelets, AF568-annexin A5 to label phosphatidylserine-exposing platelets, and AF647-fibrinogen to stain formed fibrin fibers. Coperfusion of blood from patients with severe FXI deficiency with CaCl _2_ /MgCl _2_ over microspots of collagen/TF for 11 minutes resulted in a substantial prolongation and suppression of fibrin formation, while this was not seen for blood from a patient with moderate FXI deficiency (FXI: 6 IU/dL) ( Fig. 3 ). Staining for platelet deposition (DiOC _6_ ) and phosphatidylserine exposure (AF568-annexin A5) at end-stage was in the same range for patients and control subjects. However, quantification of microscopic images indicated that, for the majority of patients, the time-to-fibrin formation was prolonged, whilst the amount of fibrin formed at end-stage was reduced, in comparison to the controls ( Fig. 4A ). These differences were most noticeable on collagen/TF microspots, but also apparent on collagen microspots, again suggesting that TF-induced triggering of the extrinsic system has a partly normalizing effect on a delayed intrinsic fibrin–thrombus formation in these patients.

Remarkably, the blood samples from two patients with severely reduced FXI levels (1 and 4 IU/dL FXI) and a history of bleeding were most reduced and prolonged in fibrin formation, along with reduced platelet deposition. Increasing the endogenous FXI level in these two blood samples with 33 IU/dL improved the time-to-fibrin formation, platelet deposition and activation, along with fibrin formation on collagen/TF microspots ( Fig. 4B ). Together, these results point to a diminished formation of platelet–fibrin thrombi under flow conditions for those patients with the most strongly reduced FXI level, and to a partial normalization of this process for FXI-deficient patients in the presence of TF.

### Integrative Comparison of Alterations in Thrombin Generation and Thromboelastometry

**Fig. 5:**
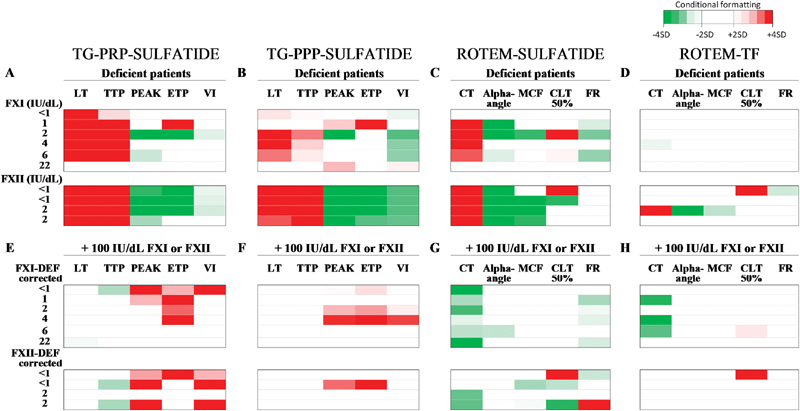
Comparative analysis of alterations in thrombin generation and thromboelastometry for individual patients. Colormaps of relevant increased ( *red* ) or decreased ( *green* ) values of indicated curve parameters of thrombin generation in PRP ( **A** and **E** ), thrombin generation in PPP ( **B** and **F** ), and thromboelastometry ( **C–D** and **G–H** ). Altered values were considered to be relevant, if outside the range of mean ± 2 SD of values from the control group ( *white* ). Borders for conditional formatting are mean ± 2 SD and mean ± 4 SD. Individual patients were ordered per line, ranking from higher to lower deficiency (factor levels indicated). Basal values ( *upper panels* ) and effects of factor repletion ( *lower panels* ) after triggering with sulfatides or TF as indicated in the figure ( **C** ). α-angle, rate of clot formation; CLT 50%, clot lysis time calculated as the interval from 50% clot formation to 50% clot lysis (s); CT, clotting time (s); ETP, endogenous thrombin potential (nM min); FR, fibrinolysis rate between lysis-onset time (LOT at 15% reduction of MCF) and lysis time (LT at 90% reduction of MCF) (% decline/min); LT, lagtime (min); MCF, maximal clot firmness (mm); PEAK (mm); PPP, platelet-poor plasma; PRP, platelet-rich plasma; SD, standard deviation; TTP, time to peak (s); VI, velocity index (nM/min).

To evaluate in more detail the changes in thrombin generation and thromboelastometry curve parameters, we produced colormaps to summarize the effects per patient (cf. residual FXI or FXII level, Fig. 5 ); arbitrarily, normal ranges were set as means ± 2 SD of the control group. These colormaps illustrate the powerful delay and reduction in sulfatide-triggered thrombin generation for both FXII- and FXI-deficient patients. Clots were formed later in the PRP and PPP samples for all FXII-deficient patients in comparison to the plasmas from FXI-deficient patients, with only consistently strong delayed curves in PRP ( Fig. 5A , B ). Interestingly, for all four FXII-deficient patients, one or more markers of extent of thrombin generation (peak height, ETP, VI) were lower than in the control samples, whilst for most of the FXI-deficient patients these markers were not influenced, and in one out of six FXI-deficient patients these parameters were higher compared to controls.

Furthermore, the colormaps show the enhanced thrombin generation profiles in plasma samples for most patients upon reconstitution with purified FXI or FXII. Regarding thromboelastometry measurements, a consistent prolongation can be seen in kinetic parameters (FXII > FXI), but only with sulfatides as a trigger. Furthermore, the tPA-dependent fibrinolysis was reduced in three out of six FXI-deficient patients ( Fig. 5C ), and was prolonged and reduced in one FXII-deficient patient (FXII <1 IU/dL), regardless of the trigger.

## Discussion

Both in physiology (hemostasis) and pathophysiology (thrombosis), the coagulation cascade is thought to be triggered by TF. Under normal conditions minute amounts of TF, present on microvesicles, cell surfaces, or as part of the so-called “hemostatic envelope,” drive a basal level of coagulation. In the present experiments we addressed the impact of FXII and FXI mainly by stimulating contact activation in blood or plasma obtained from patients with a congenital deficiency in either factor, versus controls.

First, in both thrombin generation and thromboelastometry measurements triggered with sulfatides, curve parameters were delayed and suppressed, as expected more in FXII than in FXI-deficient patients, when compared to the controls. Normalization of FXI or FXII factor levels in both PRP and PPP in the intrinsic TG revealed normalization of the lagtime. Previously reported differences between FXI-deficient patients and controls were also determined from curves of TF-stimulated thrombin generation in PRP. Also, the prolonged lagtimes and reduced peaks in thrombin generation in bleeders versus nonbleeders and controls were observed upon triggering with TF most obvious in TG with CTI-treated PRP. 33 34 In contrast, Zucker et al found no difference in TF-triggered TG measurements in FXI-deficient CTI-treated PPP. 35

Remarkably, upon normalization of the factor level by adding purified FXI or FXII, some patients showed a higher thrombin generation (increased ETP and/or peak) than the controls ( Fig. 5E , F ). This increase was more pronounced in PRP than in PPP. This result indicates that the TG process (under stasis) can be enhanced beyond normal. In contrast, such enhancement was not seen in thromboelastometry experiments, suggesting that the rate of fibrin formation was already maximized under “normal” conditions. These findings confirm that additional thrombin is formed after clot formation. The amount of thrombin formed after fibrin formation exceeds the amount of thrombin needed for fibrin formation. 36 These high TG data are in line with clinical experience reporting hypercoagulability and thrombotic complications in some patients with FXI deficiency when they receive replacement therapy with high-dose FXI. 37 Moreover, epidemiological data show that high levels of FXI are a risk factor for venous thrombosis. 38 39

In the presence of platelets, lagtime and time to peak in thrombin generation in FXI deficiency were more prolonged as compared to added phospholipids, which may indicate the importance of platelets in establishing a functional intrinsic system. Data from Kossmann et al 18 showed that FXI activation occurs on platelets in a GPIb-V-IX-dependent manner, hence the degree of platelet activation may be a determinant in these reactions. From our data, the degree of platelet activation cannot be derived after triggering contact activation, although it can be assumed to play a role, given the formation of thrombin, a potent platelet activator.

Second, clot formation and the fibrinolytic aspects were tested in ROTEM experiments in the presence of tPA. Lower levels of FXII or FXI delayed clot formation in thromboelastometry upon triggering with sulfatides. In addition, the formed clots were smaller in FXII-deficient patients. Fibrinolysis did not differ between patient and controls; however, when we evaluated the patients individually, we observed that half of the FXI-deficient patients had a reduced FR. These same patients also had a decreased α-angle indicating impairment in the build-up and breakdown of the clot. After the addition of purified FXI, the α-angle was normal in all patients; however, more than half of FXI-deficient patients had a reduced FR with normal CLT 50%. This indicates that the breakdown of the clot was impaired in the last part of fibrinolysis. In PRP and to a lesser extend in PPP, we observed an increased ETP in FXI-deficient patients after reconstitution with purified FXI. The formation of thrombin inside the clot protects against fibrinolysis possibly explaining this reduction in FR. 36 These data support a contributory role of FXII and FXI in clot formation upon contact activation, as well as a role in clot lysis. Thromboelastography has been used to investigate coagulation in FXI-deficient patients. However, these publications used the commercial INTEM reagent which uses ellagic acid (which is not a physiological trigger) to activate FXII or a low concentration of TF; fibrinolysis was often not investigated and plasma instead of whole blood has been used. 40 41 42

The net effect of FXI and FXII on the balance between clot formation and breakdown is complex. Earlier studies in humans show an effect of FXII on fibrinolysis, with subjects deficient in FXII having a diminished clot lysis. 43 Furthermore, activation of coagulation by FXII/FXI drives thrombin formation which in turn is an important determinant of clot strength and fibrinolysis, among others by activation of FXIII and TAFI. 44 Part of this complexity may also explain the nonlinear effects of FXII activity in relation to arterial thrombotic events.

Replenishing of factors shortened clot formation upon FXI correction and CLT (CLT 50%) measured to 50% degradation of the clot was normalized.

Upon TF activation (in CTI-containing blood), the thromboelastometry parameters showed less remarkable differences between FXII- or FXI-deficient patients and controls. These results are in line with a previous study where upon TF triggering using thromboelastometry no significant difference in parameters was seen between FXI-deficient patients when measured in CTI-treated whole blood. 41 Only in one FXII-deficient patient (FXII <1 IU/dL) fibrinolysis was delayed and decreased in thromboelastometry, although more pronounced after sulfatide triggering. In this patient sample, higher tPA levels were needed to lyse the clot formed. Here, it is relevant to note that in a case report another severe FXII-deficient patient with diminished fibrinolysis was reported who experienced a myocardial infarction. 45

Third, under flow conditions using whole blood samples, triggered with collagen ± TF to stimulate fibrin–thrombus formation, in the majority of FXI-deficient patients the time-to-fibrin formation was delayed and fibrin formed was reduced; however, there was hardly a difference in platelet deposition and phosphatidylserine exposure. Previous work has shown a key role of immobilized collagen in enhancing coagulation via the FXI/FXII pathway. 46 47 Zhu et al also found in flow experiments where FXI antibodies 14E11 or O1A6 were added to blood of healthy individuals efficiently abolished thrombin and fibrin generation without affecting platelet deposition on the collagen surface. 48 They did not measure phosphatidylserine exposure. Our result was confirmed with confocal fluorescence images and interestingly also indicates that the degree of FXI deficiency is important, since a severe FXI-deficient patient (<1 IU/dL) showed an impaired thrombus and fibrin formation when compared with a moderate FXI-deficient patient (6 IU/dL) and a control. Addition of purified FXI in the blood samples of two FXI-deficient patients marked as severe bleeders improved thrombus formation and activation of platelets in a flow experiment on collagen/TF microspots. Although we could only assess one severe FXII-deficient patient in these flow experiments, both in the absence or presence of TF the platelet deposition was diminished (data not shown). The available data indicate an important role for the thrombin–FXI feedback loop involving platelets, in TF/collagen-dependent thrombus formation under flow. This also strengthens the notion that FXI is involved in the initial hemostatic clot forming process, which helps to explain the mild bleeding disorder in FXI-deficient subjects. As established before, deficiency in FVIII or FIX (associated with major bleeding episodes) caused a much larger suppressing effect on the fibrin formation under flow. 49

The main limitations of this study concern the low number of subjects available in one center, also due to the low prevalence of these deficiencies. Another limitation is that the degree of variation in test outcomes does not allow for definitive conclusions on associations between coagulation responses on the one hand and bleeding tendency on the other hand.

In conclusion, in thrombin generation tested in sulfatide-activated plasma, or sulfatide-activated blood tested in ROTEM, deficiency of FXI or FXII had a substantial effect on thrombin and clot formation respectively, including a remarkable and late occurring delay in clot lysis, in most FXI-deficient patients. Furthermore, clot formation induced by TF/collagen was strongly determined by the residual level of FXI in the patient's blood and was corrected by the addition of purified FXI. The residual FXI level does not always determine the bleeding severity. Therefore, the impact of a congenital FXI deficiency on bleeding tendencies and treatment of individuals with thrombotic disorders with inhibitors of FXIa or FXIIa should be further investigated to better understand the impact on hemostasis and the potential bleeding risk associated with such interventions.
