# Supplementary material for: Impact of Deficiency of Intrinsic Coagulation Factors XI and XII on Ex Vivo Thrombus Formation and Clot Lysis
Source: TH Open. 2019 Sep 10;3(3):e273–85. doi: 10.1055/s-0039-1693485 (PMC6736668; doi:10.1055/s-0039-1693485)
Supplement: Supplementary file 1 — Supplementary Material [file 10-1055-s-0039-1693485-s190016.pdf]

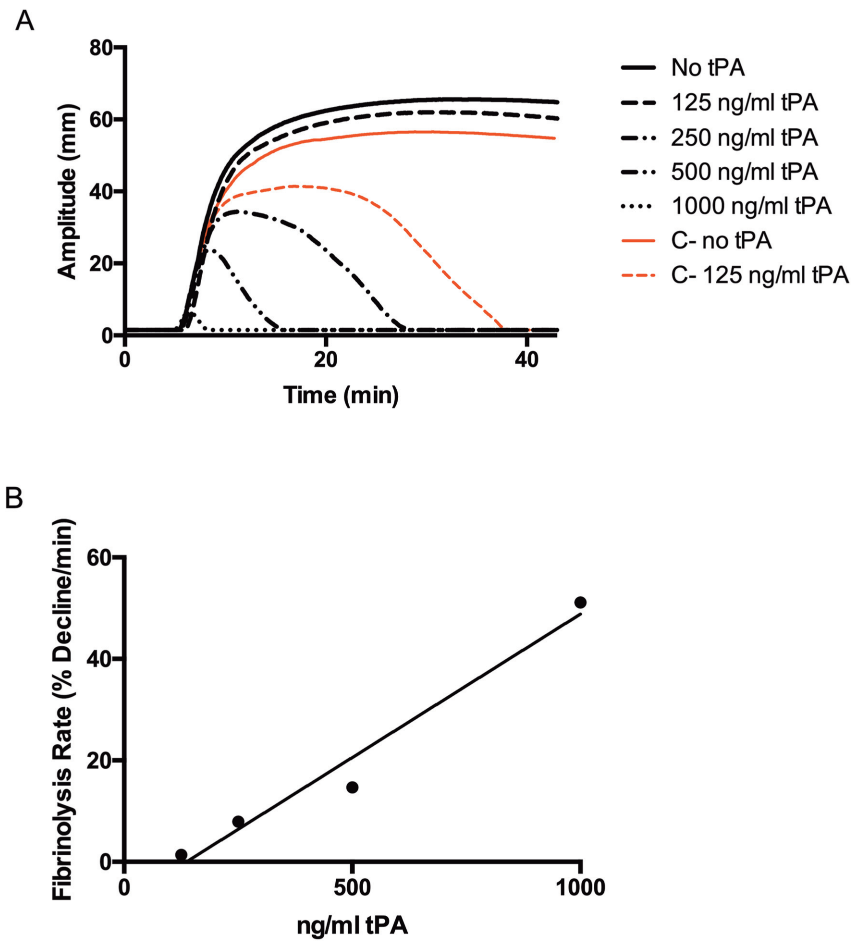

Supplementary Fig. S1 Normalization of low-rate fibrinolysis in FXII-deficient blood by increased tPA concentration. ROTEM was used to assess elasticity in whole blood from a FXII-deficient patient (<1 IU/dL, *black*) and control subject (C-) (*red*) triggered with 15 pmol/L TF and 16 mmol/L $CaCl_2$. Blood samples were pretreated with different amounts of tPA (125–1,000 ng/mL), as indicated. (**A**) Plots of thromboelastic amplitude in time (clot firmness). (**B**) Plot of fibrinolysis rate at different tPA concentrations. Fibrinolysis rate was calculated from the decay curve parameters, as indicated in the Methods. TF, tissue factor; tPA, tissue-type plasminogen activator.
